# MicroRNA-33a-mediated downregulation of Pim-3 kinase expression renders human pancreatic cancer cells sensitivity to gemcitabine

**DOI:** 10.18632/oncotarget.3885

**Published:** 2015-05-08

**Authors:** Chen Liang, Xian-Jun Yu, Xiao-Zhong Guo, Meng-Hong Sun, Zhen Wang, Yao Song, Quan-Xing Ni, Hong-Yu Li, Naofumi Mukaida, Ying-Yi Li

**Affiliations:** ^1^ Cancer Research Institute, Fudan University Shanghai Cancer Center, Department of Oncology, Shanghai Medical College, Fudan University, Shanghai, China; ^2^ Department of Pancreas and Hepatobiliary, Pancreatic Cancer Institute, Fudan University Shanghai Cancer Center, Department of Oncology, Shanghai Medical College, Fudan University, Shanghai, China; ^3^ Department of Gastroenterology, General Hospital of Shenyang Military Area, Shenyang, Liaoning, China; ^4^ Department of Pathology, Fudan University Shanghai Cancer Center, Department of Oncology, Shanghai Medical College, Fudan University, Shanghai, China; ^5^ Division of Molecular Bioregulation, Cancer Microenvironment Research Program, Cancer Research Institute, Kanazawa University, Kanazawa, Japan

**Keywords:** miR-33a, serine/threonine kinase, tumor suppressor, chemoresistance, pancreatic cancer

## Abstract

Pancreatic ductal adenocarcinoma (PDAC) is one of the most lethal cancers, with less than 5% of patients surviving 5 years beyond diagnosis. Systemic therapies, particularly gemcitabine, have a modest clinical benefit, but chemoresistance limits their efficacy. Here, we demonstrate that plasma miR-33a levels positively correlated with miR-33a levels in tumor tissues of patients with PDAC and are a good prognostic indicator of overall survival. Overexpression of miR-33a inhibited tumor cell proliferation and increased the chemosensitivity to gemcitabine both *in vitro* and *in vivo*. Moreover, miR-33a targets Pim-3 directly in PDAC. Pim-3 expression was a prognostic indicator related to poor survival in pancreatic cancer patients. Plasma miR-33a levels were significantly lower in pancreatic cancer patients with high Pim-3 protein expression than in healthy controls. Furthermore, overexpression of miR-33a in pancreatic cancer cell lines suppressed Pim-3 expression, leading to downregulation of the AKT/Gsk-3β/β-catenin pathway. Overall, these results indicate that miR-33a functions as a tumor suppressor that downregulates Pim-3 kinase expression to inhibit both pancreatic tumor growth and gemcitabine resistance via the AKT/β-catenin pathway. Hence, detection of plasma miR-33a may be a simple and convenient method of predicting therapeutic responses.

## INTRODUCTION

Pancreatic ductal adenocarcinoma (PDAC) is one of the most deadly human malignant neoplasms and the fourth leading cause of cancer-related deaths in the United States [1]. With an overall 5 year survival rate <5% and a median survival of <1 year, pancreatic cancer has an extremely poor prognosis [2]. Gemcitabine, which has been available for more than a decade, is the only first-line chemotherapeutic agent used for the palliative treatment of patients with PDAC [3]. However, the tumors in a substantial number of patients are already (or rapidly become) chemoresistant to gemcitabine [2]. Recent studies identified several chemoresistant mechanisms associated with the metabolism and molecular targets of gemcitabine [4, 5]. Combining gemcitabine with a targeted therapeutic strategy may be beneficial for the treatment of gemcitabine-refractory pancreatic cancer.

MicroRNAs (miRNAs) are a class of single-stranded, small, noncoding RNAs that regulate gene expression by interfering with specific mRNAs at the post-transcriptional level [6]. By regulating the expression of cancer-specific genes, miRNAs can function as either oncogenes or tumor suppressors [7, 8]. Dysregulated miRNAs are associated with PDAC proliferation, invasion, chemosensitivity, and prognosis [9]. A more complete understanding of the unique patterns of dysregulated miRNAs in PDAC may identify potential therapeutic targets or molecular biomarkers and improve tumor diagnosis or predictions of therapeutic responses.

Previous studies examined the contributions of miRNAs to pancreatic carcinogenesis and diagnosis. For example, let-7 expression is significantly lower in pancreatic cancer cells than in normal acinar pancreatic tissue; restoration of let-7 expression inhibits cell proliferation, K-ras expression, and mitogen-activated protein kinase activation, but does not inhibit tumor growth after intratumoral gene transfer [10]. Moreover, miR-34 restores the tumor-suppressive function of p53 in p53-deficient human pancreatic cancer cells by modulating downstream Notch signaling and Bcl-2; it also plays an important role in self-renewal of pancreatic cancer cells [11]. By contrast, miR-21 is upregulated in pancreatic cancer cells, where it targets PTEN, PDCD4, TPM1, and TMP3, leading to the inhibition of apoptosis [12].

Pim-3, a member of the proto-oncogene Pim family, exhibits serine/threonine kinase activity [13] and has high sequence similarity to Pim-1 and Pim-2 [14]. We previously showed that Pim-3 protein expression is increased in premalignant and malignant lesions of endoderm-derived organs such as the pancreas, colon, liver, and stomach, whereas it is expressed at barely detectable levels in these normal adult organs [15]. Moreover, Pim-3 regulates tumor cell proliferation, survival, and apoptosis during early carcinogenesis [16, 17]. A recent study demonstrated that inhibiting Pim-3 kinase sensitizes pancreatic cancer cells to gemcitabine [18]. However, the precise mechanisms by which signaling responses contribute to Pim-3-mediated pancreatic carcinogenesis and chemoresistance remain to be elucidated.

Here, we showed that miR-33a acts as an important suppressor of human pancreatic cancer by directly regulating Pim-3 expression at the post-transcriptional level. Overexpression of miR-33a led to a marked downregulation of Pim-3 expression, thereby inhibiting AKT/Gsk-3β/β-catenin signaling, which in turn reduced cell proliferation and increased the chemosensitivity of pancreatic cancer cells to gemcitabine both *in vitro* and *in vivo*. The results suggest that the plasma level of miR-33a may be a valuable biomarker and an important prognostic factor for human pancreatic cancer.

## RESULTS

### Plasma miR-33a expression is positively correlated with tissue miR-33a expression and an improved prognosis for patients with pancreatic cancer

**Figure 1:**
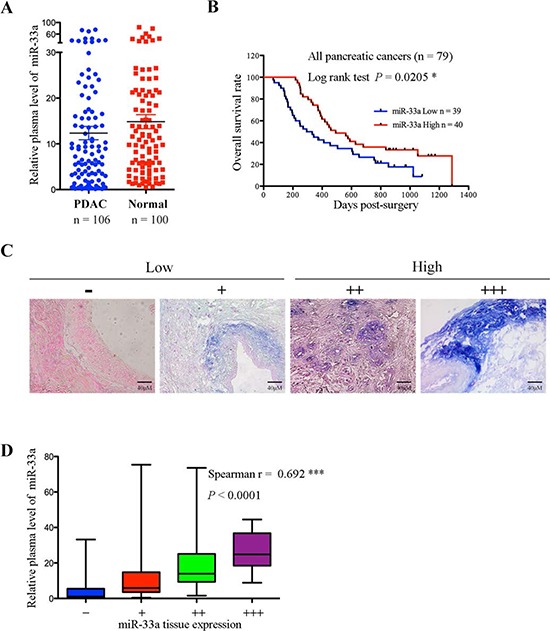
MiR-33a expression is reduced in plasma and tumor tissues from pancreatic cancer patients and is a prognostic indicator for pancreatic cancer **A.** Comparison of miR-33a expression levels in plasma from pancreatic cancer patients and healthy controls. The expression levels of miR-33a were determined by qRT-PCR and normalized to those of cel-miR-39. The horizontal line represents the median value, and the error bars indicate the SEM. **B.** Overall survival analysis based on the expression levels of miR-33a in the plasma. **C.** miR-33a expression in serial sections of human pancreatic cancer tissues, as analyzed by *in situ* hybridization. The scoring criteria are described in the Materials and Methods. **D.** Positive correlation between the plasma and tumor tissue miR-33a levels in 106 PDAC patients (Spearman's *r* = 0.692, ***P < 0.001). The ends of the whiskers represent the 1^st^ and 99^th^ percentiles.

MiR-33a expression is associated with an improved prognosis for PDAC [19]. Therefore, we first examined miR-33a levels in the plasma of 106 PDAC patients and 100 healthy controls. We found that plasma miR-33a levels were higher in healthy controls than in PDAC patients (Figure 1A). Low plasma miR-33a levels were significantly associated with tumor size (*P* = 0.0381; Table 1). A Kaplan-Meier analysis indicated that low levels of plasma miR-33a led to a significant reduction in the overall survival of 79 PDAC patients (Figure 1B). Moreover, *in situ* hybridization detected visible and gradable expression of miR-33a in tissue samples from the 106 PDAC patients (Figure 1C). Also, miR-33a levels in the plasma positively correlated with miR-33a levels in the pancreatic tumors of the 106 patients (Figure 1D).

**Table 1:** Correlation between plasma miR-33a expression and the clinicopathological features of 106 PDAC specimens

	No. of cases	No. of patients (%)		*P*-value
		miR-33a High	miR-33a Low	
**Age**				
≤62 years	54	27 (50.0%)	27 (50.0%)	1.0000
>62 years	52	26 (50.0%)	26 (50.0%)	
**Gender**				
Male	57	34 (59.6%)	23 (40.4%)	0.0509
Female	49	19 (38.8%)	30 (61.2%)	
**Tumor Location**				
Head	54	27 (50.0%)	27 (50.0%)	1.0000
Body and Tail	52	26 (50.0%)	26 (50.0%)	
**Tumor Size**				
≤4 cm	71	41 (57.7%)	30 (42.3%)	0.0381*
>4 cm	35	12 (34.3%)	23 (65.7%)	
**Nodal Metastasis**				
N_0_	62	31 (50.0%)	31 (50.0%)	1.0000
N_1_-N_3_	44	22 (50.0%)	22 (50.0%)	
**Tumor Differentiation**				
Well or Moderate	72	37 (51.4%)	35 (48.6%)	0.8354
Poor	34	16 (47.1%)	18 (52.9%)	
**TNM Stage**				
I and II	83	43 (51.8%)	40 (48.2%)	0.6381
III and IV	23	10 (43.5%)	13 (56.5%)	

### MiR-33a inhibits cell proliferation and increases the chemosensitivity of human pancreatic cancer cells to gemcitabine both *in vitro* and *in vivo*

**Figure 2:**
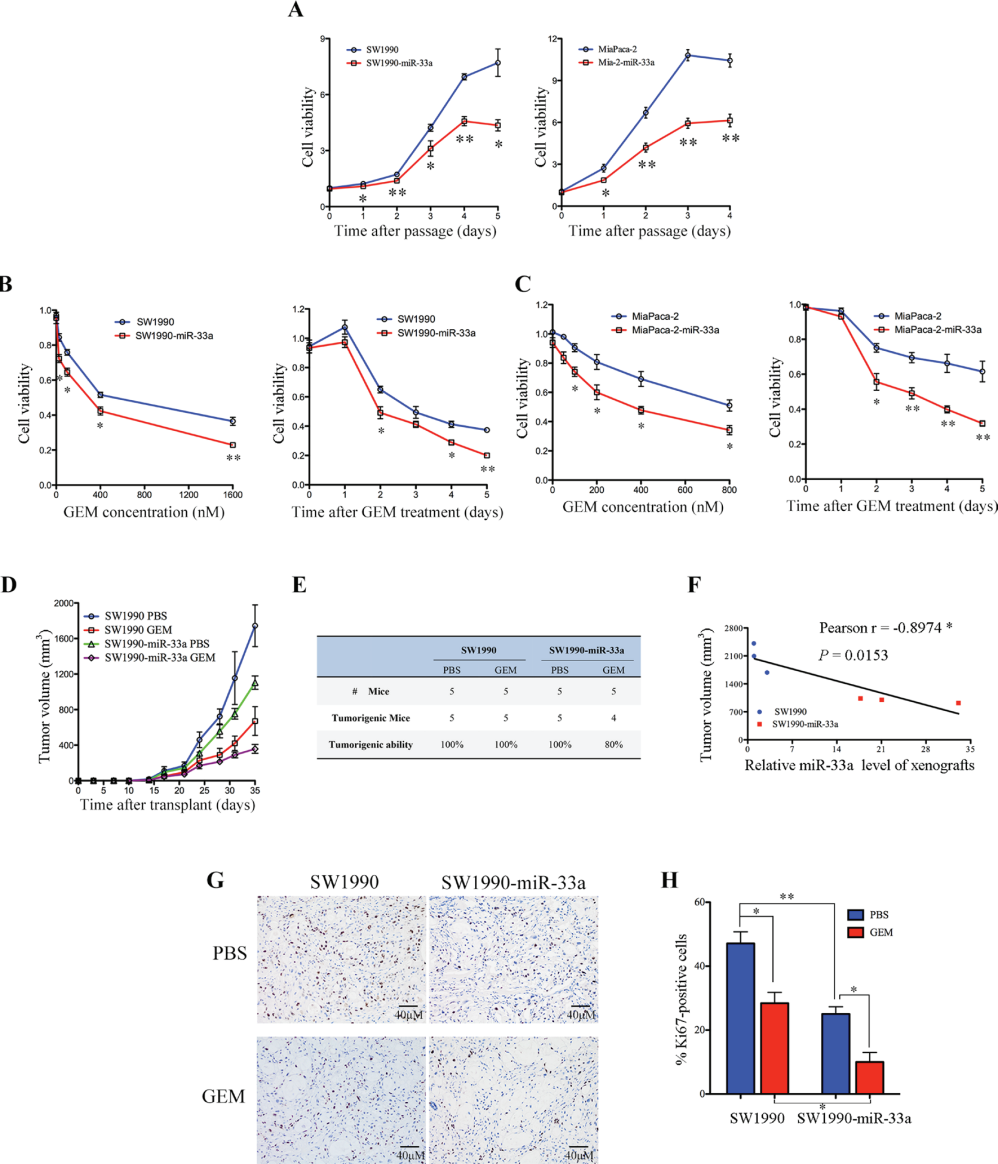
Overexpression of miR-33a increases the chemosensitivity of human pancreatic cancer cells to gemcitabine both *in vitro* and *in vivo* **A.** The effects of stable overexpression of miR-33a on pancreatic cancer cell viability. The cell numbers were determined at the indicated time intervals using Cell Counting Kit-8 reagent and were normalized to those at time 0. Data are expressed as the mean +/− SD of five replicates. The experiments were repeated three times, and representative results are shown. *P > 0.05 and **P > 0.01. **B, C.** The effects of gemcitabine (GEM) on the viability of SW1990-miR-33a (B) and Mia-2-miR-33a (C) cells and their parental cells. The cells were treated with increasing concentrations of GEM for 48 h (B and C, left panel) or with 200 nM GEM [SW1990 cells] (B, right panel) or 350 nM GEM [MiaPaca-2 cells] (C, right panel) for 0–5 days. Cell numbers were determined using Cell Counting Kit-8 as described in the Materials and Methods. The results are expressed as the mean +/− SD of five replicates from three independent experiments. *P > 0.05 and **P > 0.01. **D.** Size of tumors in mice that received subcutaneous implants of SW1990-miR-33a cells or parental cells. The mice were treated with GEM or PBS (control), and tumor growth was measured twice per week. The data are expressed as the mean +/− SD. **E.** Tumor formation in the mice described in (D) on Day 35. **F.** Negative correlation between the volume of the xenograft tumors and miR-33a expression (Pearson's *r* = −0.8974, *P* = 0.0153). **G.** Immunohistochemical analyses of Ki67-positive cells in tumor xenograft samples from mice treated with GEM or PBS. Magnification, ×400. **H.** The number of Ki67-positive cells per field (five randomly selected visual fields) at 400× magnification. Data are expressed as the mean +/− SD of three samples per group. *P > 0.05, **P > 0.01, and ***P > 0.001.

Since the poor prognosis for PDAC is due in part to chemoresistance [2], we next examined the relationship between miR-33a and gemcitabine resistance *in vitro*. Stable expression of miR-33a in the SW1990 and MiaPaca-2 pancreatic cancer cell lines (SW1990-miR-33a and Mia-2-miR-33a) led to a significant reduction in growth (Figure 2A) and in the IC_50_ value of gemcitabine for SW1990 and MiaPaca-2 cells (Table 2). In addition, overexpression of miR-33a increased the sensitivity of both cell lines to gemcitabine in a dose- and time-dependent manner (Figure 2B and 2C). To assess the function of miR-33a *in vivo*, SW1990 or SW1990-miR-33a cells were subcutaneously implanted into nude mice. Tumor growth in the gemcitabine-treated SW1990-miR-33a group was slower than that in the PBS-treated and gemcitabine-treated SW1990 groups (Figure 2D). Furthermore, overexpression of miR-33a in gemcitabine-treated SW1990 cells resulted in fewer tumors in these mice (Figure 2E). Similar results were obtained using mice injected with MiaPaca-2 and Mia-2-miR-33a cells (Supplementary Figure 1A and 1B). There was a significant negative correlation between xenograft size and miR-33a expression (Figure 2F; *r* = −0.8974, *P* = 0.0153), indicating that miR-33a negatively regulates tumor volume by increasing the chemosensitivity of pancreatic cancer cells to gemcitabine. In addition, immunohistochemical analyses revealed that stable expression of miR-33a acted synergistically with gemcitabine to reduce the number of proliferating Ki67-positive cells (Figure 2G and 2H).

**Table 2:** The IC_50_ values for gemcitabine and the resistance indices of pancreatic cancer cells

Cell lines	IC_50_	RI	Cell lines	IC_50_	RI
SW1990	502.3 ± 39.7 nM	215 ± 19.73	MiaPaca-2	987.2 ± 82.7 nM	173.1 ± 7.15
SW1990-miR-33a	204.5 ± 19.2 nM**		Mia-2-miR-33a	336.5 ± 41.0 nM**	
SW1990-res	106.5 ± 17.2 μM		Mia-2-res	168.6 ± 3.8 μM	
SW1990-res-miR-33a	28.21 ± 3.3 μM^#^		Mia-2-res-miR-33a	61.9 ± 10.1 μM^###^	

**Abbreviations:** IC_50_: 50% inhibitory concentration; RI: Resistance index.

** *P* > 0.01 versus the parental cells;

# *P* > 0.05

### *P* > 0.001 versus the resistant cells.

### MiR-33a reverses the chemoresistance of human pancreatic cancer cells to gemcitabine both *in vitro* and *in vivo*

**Figure 3:**
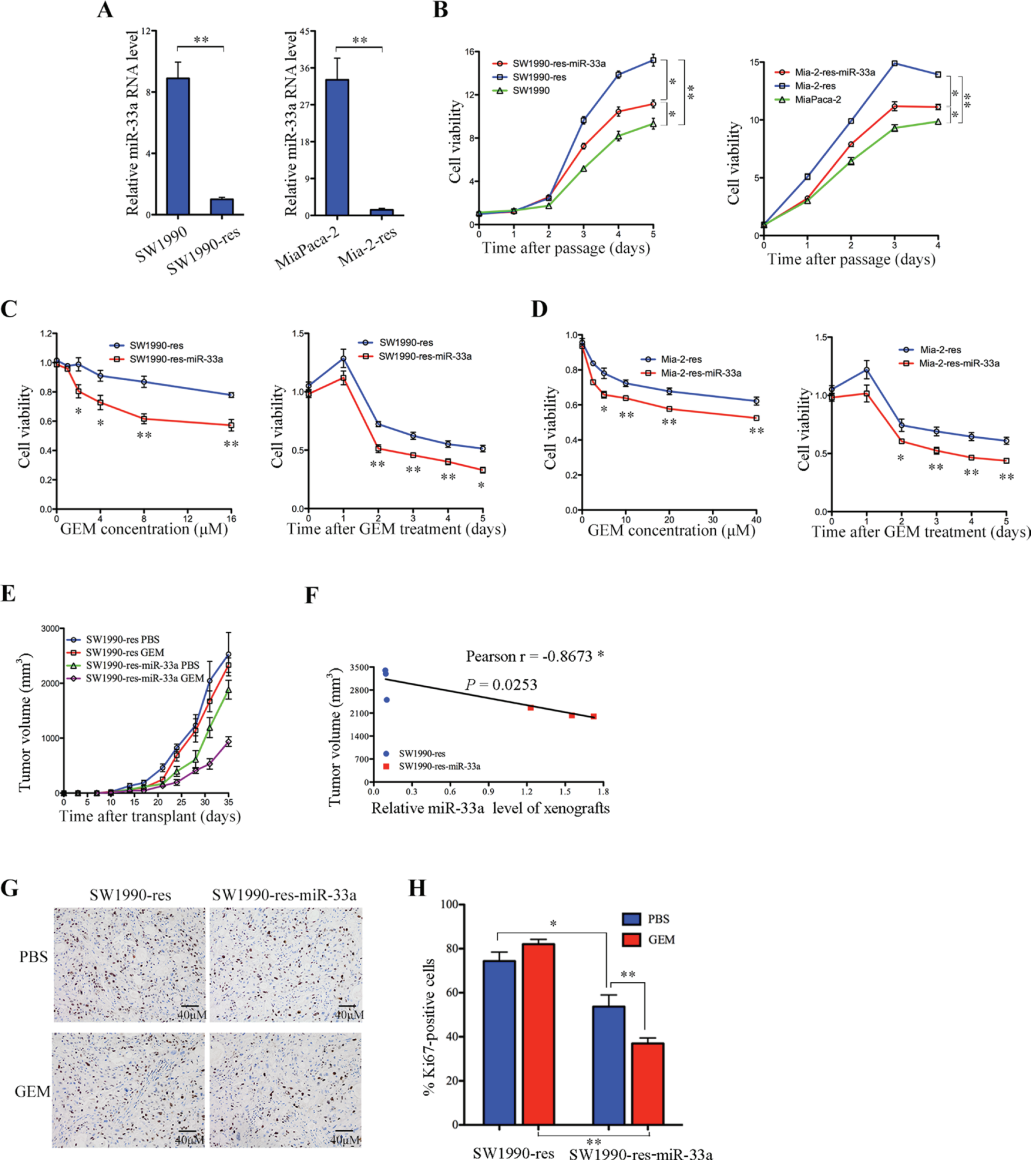
Overexpression of miR-33a reverses the chemoresistance of pancreatic cancer cells to gemcitabine both *in vitro* and *in vivo* **A.** The expression levels of miR-33a in the indicated resistant and parental cell lines, as determined by qRT-PCR. Data are expressed as the mean +/− SD of three independent experiments. **P > 0.01. **B.** The effects of stable miR-33a expression on the viability of gemcitabine-resistant pancreatic cancer cells (Mia-2-res). Cell numbers were determined as the indicated time points using the Cell Counting Kit-8 reagent, and the ratios were compared with that at time 0. Data are expressed as the mean +/− SD of five replicates. All experiments were repeated three times, and representative results are shown. *P > 0.05 and **P > 0.01. **C, D.** The effects of gemcitabine (GEM) on the viability of SW1990-res-miR-33a (C) and Mia-2-res-miR-33a (D) cells and their parental resistant cell lines. The cells were treated with increasing concentrations of GEM for 48 h (C and D, left panels) or with 30 μM GEM [SW1990-res] (C, right panel) or 60 μM GEM [Mia-res cells] (D, right panel) for 0–5 days. The cell numbers were determined using Cell Counting Kit-8, as described in the Materials and Methods. The results are expressed as the mean +/− SD of five replicates from three independent experiments. *P > 0.05 and **P > 0.01. **E.** Size of tumors in mice that received subcutaneous implantations of SW1990-res-miR-33a cells or parental resistant cells. The mice were treated with GEM or PBS (control), and tumor growth was measured twice per week. Data are expressed as the mean +/− SD. **F.** Negative correlation between the volume of xenograft tumors and the miR-33a expression level (Pearson's *r* = −0.8673, *P* = 0.0253). **G.** Immunohistochemical analyses of Ki67-positive cells in tumor xenograft samples from mice treated with GEM or PBS. Magnification, ×400. **H.** The number of Ki67-positive cells per field (five randomly selected visual fields) at 400× magnification. Data are expressed as the mean +/− SD of three samples per group. *P > 0.05 and **P > 0.01.

To confirm the hypothesis that miR-33a is involved in chemoresistance of PDAC, we established stable SW1990 and MiaPaca-2 cell lines that were resistant to gemcitabine (SW1990-res and Mia-2-res); the resistance indices (RI) for these cell lines were 215 (95% CI, 195.27–234.73) and 173.1 (95% CI, 165.95–180.25), respectively (Table 2). Notably, the resistant cells expressed low levels of miR-33a (Figure 3A), and their growth rates were higher than those of the corresponding parental cell lines (Figure 3B). To determine whether overexpression of miR-33a reverses gemcitabine resistance, miR-33a was stably expressed in SW1990-res and Mia-2-res cells to generate SW1990-res-miR-33a and Mia-2-res-miR-33a cells, respectively. We observed that overexpression of miR-33a led to a significant reduction in the growth rate of the resistant cell lines (Figure 3B). Moreover, overexpression of miR-33a reduced the IC_50_ value of gemcitabine towards the resistant cells (Table 2) and reduced cell viability in both a dose- and time-dependent manner (Figure 3C and 3D). Furthermore, overexpression of miR-33a in SW1990-res cells repressed tumor growth and reduced resistance to gemcitabine *in vivo* (Figure 3E). Similarly, overexpression of miR-33a in Mia-2-res cells reduced the tumor mass (Supplementary Figure 2). There was a significant negative correlation between xenograft size and miR-33a expression in mice injected with SW1990-res cells (Figure 3F; *r* = −0.8673, *P* = 0.0253). After gemcitabine treatment, the tumor shrinkage ratio was most marked in mice that received xenografts of non-resistant cells stably overexpressing miR-33a (Supplementary Figure 3). In addition, stable expression of miR-33a reduced the number of proliferating Ki67-positive cells in SW1990-res tumors, and acted synergistically with gemcitabine to inhibit this parameter (Figure 3G and 3H).

### MiR-33a directly targets the 3′UTR of *Pim-3* in pancreatic cancer cell lines

**Figure 4:**
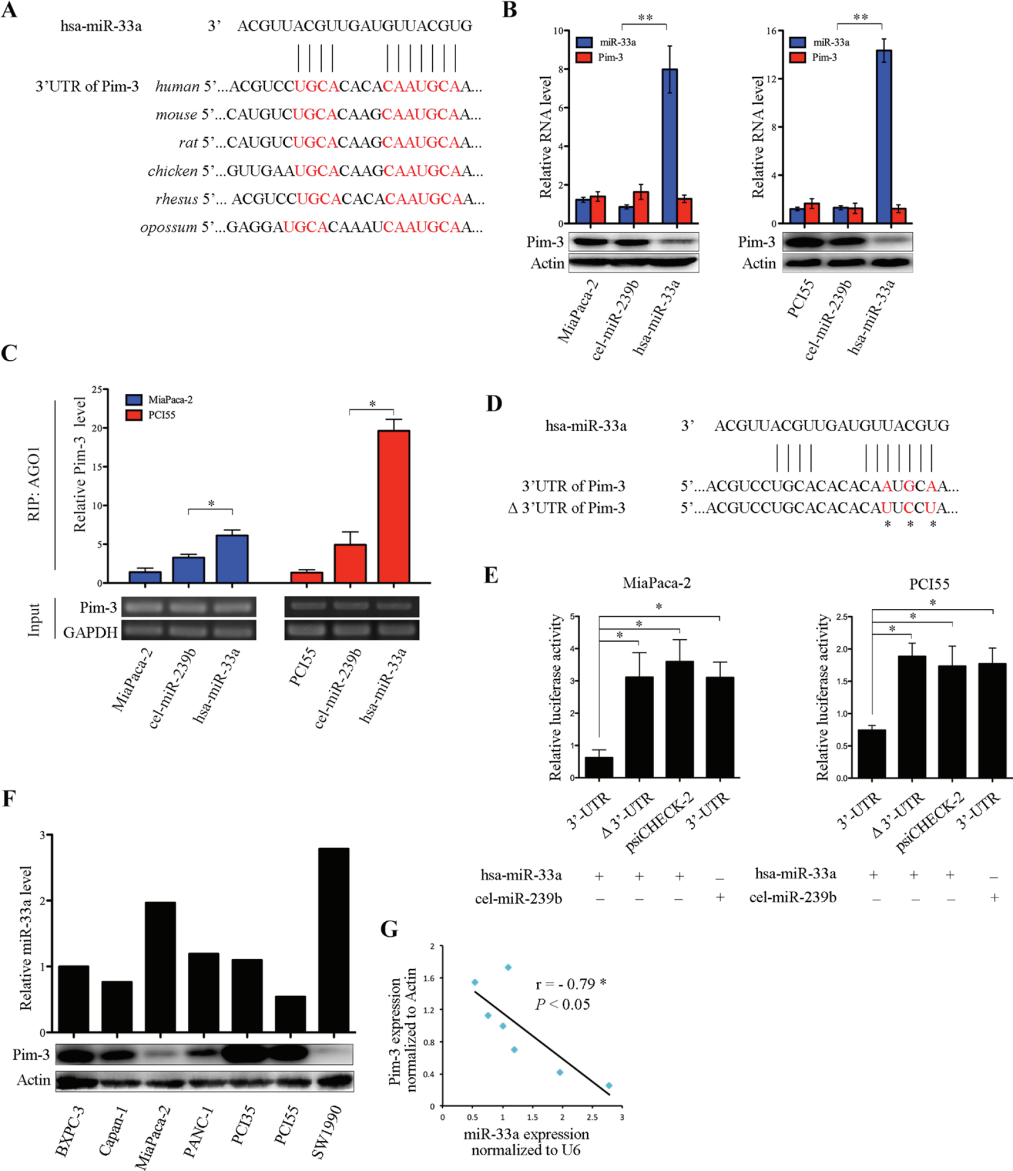
miR-33a suppresses Pim-3 expression by directly targeting its 3′UTR **A.** Alignment of the miR-33a target sequences within the 3′UTR of *Pim-3* from different vertebrates. The seed match sequences are shown in red. **B.** qRT-PCR analyses of miR-33a and *Pim-3* mRNA levels (top panel), and western blot analyses of Pim-3 protein levels (bottom panel) in MiaPaca-2 and PCI55 cells transfected with miR-33a (hsa-miR-33a) or with negative control (cel-miR-239b) mimics. The experiments were repeated three times, and representative results are expressed as the mean +/− SD. **P > 0.01. **C.** RIP analyses of *Pim-3* mRNA bound to AGO1 in MiaPaca-2 and PCI55 cells transfected with hsa-miR-33a or cel-miR-239b. Semi-quantitative PCR analyses of the input material (without immunoprecipitation) are also shown. GAPDH was used as a loading control. The experiments were repeated three times, and representative results are expressed as the mean +/− SD. *P > 0.05. **D.** Sequences of the wild-type and mutated (Δ) *Pim-3* 3′UTRs in the luciferase reporter constructs used in **E.** The asterisks indicate mutations in the miR-33a seed region. **E.** Relative luciferase activities of reporter constructs (psiCHECK-2) containing the wild-type or mutated *Pim-3* 3′UTR. MiaPaca-2 and PCI55 cells were co-transfected with reporter construct and hsa-miR-33a or cel-miR-239b. Renilla luciferase activity was determined 48 h after transfection and was normalized to that of constitutive firefly luciferase. The experiments were repeated three times, and representative results are expressed as the mean +/− SD. *P > 0.05. **F.** The expression levels of miR-33a and Pim-3 protein in various human pancreatic cancer cell lines, as determined by qRT-PCR (top panel) and western blot (bottom panel) analysis, respectively. The expression level of small nuclear RNA U6 was used to normalize the qRT-PCR results, and β-actin was used to ensure equal protein loading. **G.** Correlation between miR-33a and Pim-3 protein expression in human pancreatic cancer cell lines (Pearson's *r* = −0.79, *P* > 0.05).

A previous study shows that miR-33a interact with Pim-1 in cancer cells [20]. Kinases belonging to the Pim family are highly homologous [14], suggesting that miRNAs that bind Pim-1 may also bind Pim-3 via a similar complementary sequence in the 3′UTR of the mRNA. To examine whether miR-33a bound to Pim-3, we performed *in silico* analyses of putative miRNA-binding sites in *Pim-3* mRNA using different algorithms (www.ebi.ac.uk; www.microrna.org; and www.targetscan.org). A computational analysis (www.targetscan.org) revealed that miR-33a bound to a highly conserved sequence within the 3′UTR of *Pim-3* that is conserved across different vertebrates (Figure 4A). Moreover, transfection of MiaPaca-2 and PCI55 pancreatic cancer cells with miR-33a mimics led to a significant reduction in the expression of Pim-3 protein, but not in *Pim-3* mRNA expression (Figure 4B).

*Drosophila* AGO1, a component of the RNA-induced silencing complex, represses translation triggered by binding of miRNAs to imperfect complementary sites in the 3′UTRs of their mRNA targets [21–23]. To determine whether miR-33a-mediated downregulation of Pim-3 requires AGO1, a RIP analysis was performed using an anti-AGO1 antibody or IgG (control). The amount of *Pim-3* mRNA associated with AGO1 was significantly higher in MiaPaca-2 and PCI55 pancreatic cancer cells transfected with miR-33a mimics than in parental cells or cells transfected with negative control mimics (cel-miR-239b) (Figure 4C). However, no *Pim-3* mRNA was detected in the IgG immunoprecipitates (data not shown). Next, to determine whether Pim-3 is a direct target of miR-33a in pancreatic cancer, PCI55 and MiaPaca-2 cells were co-transfected with the miRNA mimics together with a luciferase reporter plasmid containing the wild-type 3′UTR of *Pim-3* or the same region harboring a mutation in the miR-33a seed sequence (Δ3′UTR; Figure 4D). Co-transfection of both cell lines with the miR-33a mimics repressed the luciferase activity of the wild-type *Pim-3* 3′UTR construct, but not that of the *Pim-3* Δ3′UTR construct or empty psiCHECK-2 vector (Figure 4E). Moreover, the luciferase activity of the wild-type *Pim-3* 3′UTR construct was not repressed by co-transfection of the cells with the cel-miR-239b mimics (Figure 4E). Furthermore, we also found that the expression levels of miR-33a in seven pancreatic cancer cell lines were negatively correlated with those of Pim-3 protein (Figure 4F and 4G; Spearman's *r* = −0.79, *P* > 0.05). Taken together, these results indicate that Pim-3 is a true target of miR-33a in pancreatic cancer cell lines.

### Pim-3 expression is negatively correlated with miR-33a expression and a poor prognosis

**Figure 5:**
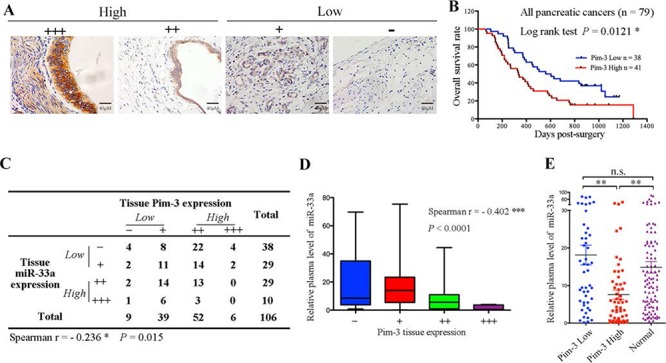
Pim-3 expression is inversely correlated with miR-33a expression **A.** Pim-3 protein expression in serial sections of human pancreatic cancer tissues, as analyzed using immunohistochemistry. The scoring criteria are described in the Materials and Methods. **B.** Overall survival analysis based on the expression levels of Pim-3 in the tumor tissue. **C.** Summary of the miR-33a and Pim-3 expression levels in 106 pancreatic ductal adenocarcinoma tissues. The negative correlation between the expression levels of miR-33a and Pim-3 was significant in human pancreatic cancer tissues (Spearman's *r* = −0.236, *P* = 0.015). **D.** The negative correlation between the plasma miR-33a levels and tumor tissue Pim-3 expression in 106 PDAC patients (Spearman's *r* = −0.402, ***P > 0.001). The ends of the whiskers represent the 1^st^ and 99^th^ percentiles. **E.** Comparison of the relative expression levels of miR-33a in plasma samples from 100 healthy controls (Normal) and 106 PDAC patients with low (*n* = 48) or high (*n* = 58) Pim-3 expression. The expression levels of miR-33a were determined by qRT-PCR and normalized to those of cel-miR-39. The horizontal line represents the median value, and the error bars indicate the SEM. ANOVA was used to determine statistical significance. **P > 0.01; n.s., not significant.

To study the relationship between miR-33a and Pim-3 in pancreatic cancer, the expression levels of Pim-3 in tissue samples from 106 patients with PDAC were determined and graded by immunohistochemistry (Figure 5A). Pim-3 protein was expressed at high levels in 54.7% (58/106) of the pancreatic cancer tissues, and Kaplan-Meier analysis indicated that high Pim-3 expression was associated with a poor prognosis in 79 pancreatic cancer patients (Figure 5B and 5C). Moreover, serial sections of the tissues revealed a negative correlation between miR-33a and Pim-3 expression (Figure 5C; *P* > 0.05). Furthermore, the miR-33a levels in plasma samples from the 106 PDAC patients were negatively correlated with the levels of Pim-3 in the pancreatic tumors (Figure 5D). Notably, plasma miR-33a levels in healthy controls were similar to those in pancreatic cancer patients with low Pim-3 expression; however, plasma miR-33a levels were significantly lower in pancreatic cancer patients with high Pim-3 expression than in controls (Figure 5E).

### Downregulation of Pim-3 by miR-33a inhibits cell proliferation and chemoresistance, in part by activating the AKT/glycogen synthase kinase 3β/β-catenin cascade

**Figure 6:**
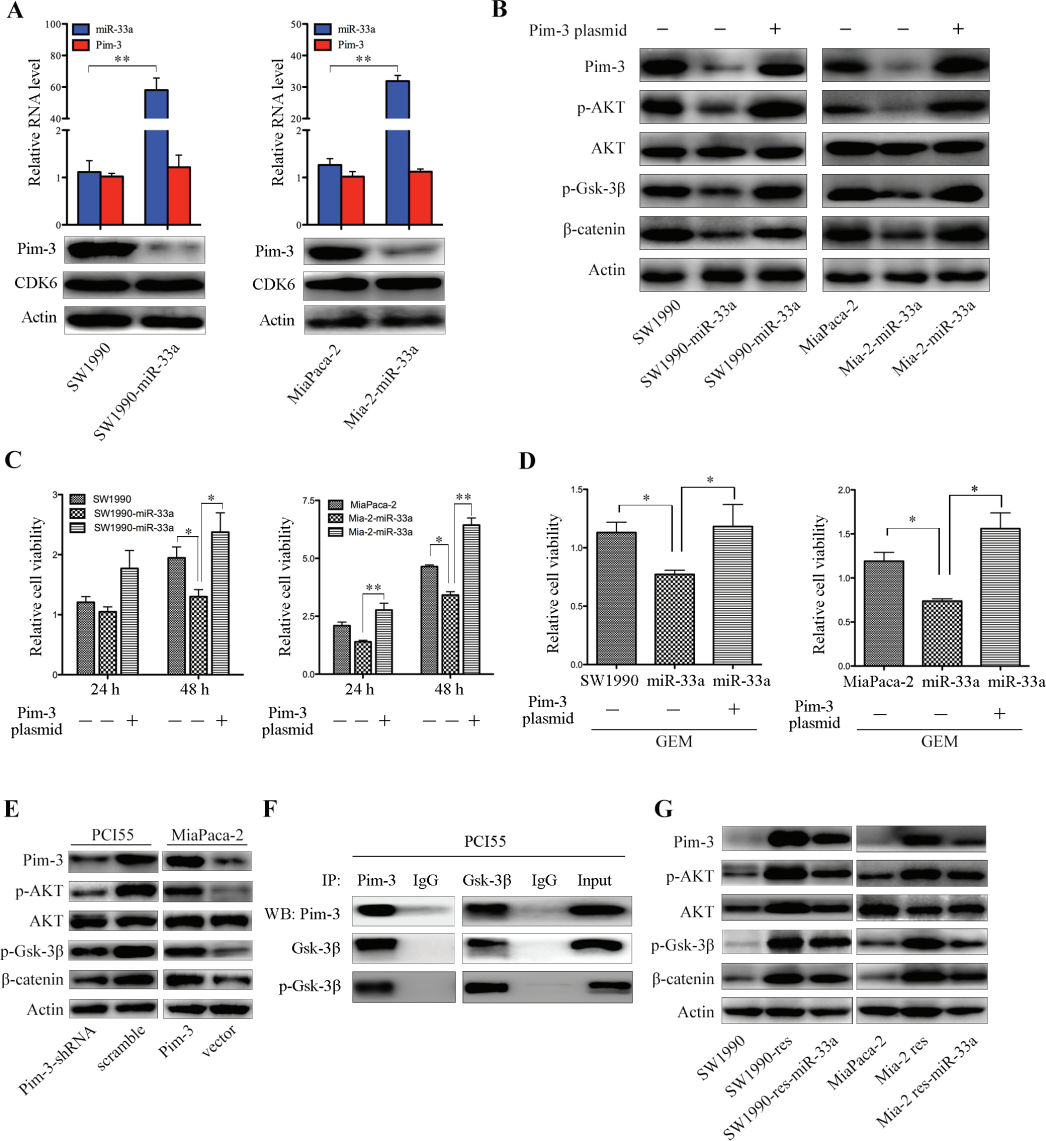
Downregulation of Pim-3 kinase by miR-33a inhibits cell proliferation and chemosensitivity, in part by regulating the AKT/Gsk-3β/β-catenin cascade **A.** qRT-PCR analyses of *Pim-3* mRNA and miR-33a levels (top panels) in SW1990 and MiaPaca-2 cells with or without stable expression of exogenous miR-33a. Data are expressed as the mean +/− SD of three independent experiments. **P > 0.01. Bottom panels show western blotting of lysates from SW1990 and MiaPaca-2 cells stably expressing miR-33a and the corresponding parental cells with the indicated antibodies. **B.** Western blotting of lysates from SW1990 and MiaPaca-2 cells stably expressing miR-33a and their parental cells with the indicated antibodies. The cells were transiently transfected with or without a pcDNA4-Pim-3 expression vector. **C.** Viability of SW1990 and MiaPaca-2 cells stably expressing miR-33a and that of their parental cells. The cells were transiently transfected with or without a pcDNA4-Pim-3 expression vector. The results are expressed as the mean +/− SD of five replicates from three independent experiments. *P > 0.05 and **P > 0.01. **D.** The effects of GEM on the viability of SW1990-miR-33a and Mia-2-miR-33a cells and that of their parental cells, which were transiently transfected with or without a pcDNA4-Pim-3 expression vector. The cells were treated with GEM [200 nM for SW1990 cells (left panel) and 350 nM for MiaPaca-2 cells (right panel)] for 48 h. The results are expressed as the mean +/− SD of five replicates from three independent experiments. *P > 0.05 and **P > 0.01. **E.** Western blot analyses of lysates from PCI55 cells stably transfected with Pim-3-specific or scrambled shRNA and MiaPaca-2 cells stably transfected with the pMEI-5-Pim-3 expression vector or an empty vector. Blots were probed with the indicated antibodies. **F.** Co-immunoprecipitation of endogenous Gsk-3β and Pim-3 in PCI55 cells. Lysates were obtained from PCI55 cells and used as an input control. The lysates were subjected to immunoprecipitation and western blot analyses with the indicated antibodies. **G.** Immunoblot analysis of the indicated proteins and phospho-proteins (p-) in lysates of SW1990 and MiaPaca-2 parental cells, GEM-resistant cells, and GEM-resistant cells stably expressing miR-33a.

Stable expression of miR-33a in SW1990 and MiaPaca-2 pancreatic cancer cells (SW1990-miR-33a and Mia-2-miR-33a) led to a reduction in the level of Pim-3 protein but not that of mRNA (Figure 6A). Although a previous study showed that miR-33a-mediated downregulation of cyclin-dependent kinase 6 (CDK6) reduces cell proliferation [20, 24], we found that overexpression of miR-33a did not downregulate CDK6 protein in SW1990 or MiaPaca-2 cells (Figure 6A), indicating that the effects on Pim-3 expression are specific to pancreatic cancer cells that we examined.

Pim-3 may be a novel regulator of AKT and Wnt/β-catenin signaling [25]. Hence, we next examined whether overexpression of miR-33a reduces β-catenin levels in SW1990 and MiaPaca-2 cells. Overexpression of miR-33a failed to reduce the total amount of AKT protein. However, the levels of endogenous Pim-3, phospho-AKT, and phospho-glycogen synthase kinase 3β (phospho-Gsk-3β) were reduced markedly under these conditions, and these changes facilitated β-catenin destruction (Figure 6B). Transient transfection of SW1990-miR-33a and Mia-2-miR-33a cells with *Pim-3* cDNA restored the amounts of phospho-AKT, phospho-Gsk-3β, and β-catenin proteins, but did not affect the total amount of AKT protein (Figure 6B). Moreover, transfection with *Pim-3* cDNA also rescued cell growth (Figure 6C) and restored resistance to gemcitabine (Figure 6D), both of which were inhibited by miR-33a overexpression. In accordance with the above results, stable expression of Pim-3 upregulated the levels of phospho-AKT, phospho-Gsk-3β, and β-catenin in MiaPaca-2 cells, whereas silencing of endogenous Pim-3 expression by stable transfection of PCI55 cells with a Pim-3-specific shRNA reduced the expression levels of these proteins markedly (Figure 6E). Pim and AKT kinases phosphorylate similar substrates and mediate a number of overlapping pathways [18, 26, 27]. Hence, we hypothesized that, like AKT [28], Pim-3 may also phosphorylate Gsk-3β to upregulate β-catenin expression. Immunoprecipitation experiments revealed that Pim-3 bound to Gsk-3β and phospho-Gsk-3β in PCI55 cells (Figure 6F), indicating a potential AKT-independent effect of Pim-3 on the β-catenin pathway.

Notably, resistant cells expressed high levels of Pim-3 protein (Figure 6G). In line with previous reports [29, 30], we found that the AKT/β-catenin signaling was activated in gemcitabine-resistant pancreatic cancer cells, and that overexpression of miR-33a reversed the increased expression of β-catenin by inhibiting Pim-3/AKT/β-catenin signaling (Figure 6G). Together with the fact that miR-33a induces the downregulation of β-catenin directly [31], or indirectly by regulating Pim-3/AKT/Gsk-3β (Figure 6E and 6F), these results reveal that the negative correlation between miR-33a expression and β-catenin expression determines gemcitabine resistance.

## DISCUSSION

The results of the *in vitro* and *in vivo* assays presented herein demonstrate that miR-33a downregulates the proto-oncogene encoding Pim-3 kinase. To the best of our knowledge, this is the first study to (i) demonstrate that plasma miR-33a levels could be a valuable biomarker and an important prognostic factor for human pancreatic cancer; (ii) provide evidence that miR-33a synergistically increases the sensitivity of PDAC cells to gemcitabine; (iii) identify Pim-3 as a direct binding target of miR-33a; and (iv) demonstrate that miR-33a downregulates Pim-3 to inhibit tumor growth and chemoresistance, in part via the AKT/Gsk-3β/β-catenin signaling cascade, in pancreatic cancer.

Circulating miRNAs enable communication between normal cells and cancer cells [32, 33], and plasma miRNA levels may reflect clinical information correlated to tumors. The data presented herein highlight the potential clinical utility of plasma miR-33a levels as a valuable biomarker that reflects the expression of miR-33a in human pancreatic cancer tissues. MiRNAs are key components of tumorigenesis, and they participate in many cellular processes, including cell proliferation and differentiation [9]. Here, we found that miR-33a suppressed both the proliferation and growth of pancreatic cancer cells. Overexpression of miR-33a was inversely correlated with both the number and size of the tumors. These observations provide the missing experimental evidence for the tumor-suppressive effect of miR-33a in human pancreatic cancer.

MiR-33a induces cisplatin-resistance in osteosarcoma cells by downregulating the transcription factor, TWIST [34]. By contrast, the results presented herein indicate that the level of miR-33a expression was significantly lower in gemcitabine-resistant cells than in parental cells. Therefore, it is possible that the gemcitabine-sensitizing effect of miR-33a is a cell- or tissue-specific phenomenon. Although miRNA expression is regulated by a number of stimuli [35], the mechanism by which miR-33a expression is inhibited after gemcitabine treatment remains unknown.

Our previous study indicated that Pim-3 is important for cell growth, apoptosis, and cell-cycle progression in human pancreatic cancer [16], although miR-124 targets Pim-3 in glioblastoma stem cells [36]. However, little is known about other miRNAs that target this kinase. The Pim kinase family consists of three members, namely, Pim-1, Pim-2, and Pim-3, which exhibit marked sequence similarity, especially within their kinase domains. Notably, a number of miRNAs regulate Pim-1 kinase [37], and bioinformatics analyses revealed that some of these miRNAs may also bind Pim-3. Based on these analyses, miR-33a was identified and confirmed as a novel miRNA that directly binds to Pim-3. The miR-33a target sequence within the 3′UTR of Pim-3 is evolutionarily conserved, suggesting that it plays an important functional role. Moreover, stable expression of miR-33a in pancreatic cancer cells led to a significant downregulation of Pim-3 protein expression and inhibited cancer cell growth both *in vitro* and *in vivo*. Furthermore, we previously showed that human pancreatic cancer cells express much lower levels of Pim-1 and Pim-2 mRNA than Pim-3 mRNA [38]. Thus, it is likely that miR-33a inhibits cell growth mainly by binding to Pim-3 in human pancreatic cancer cells.

A recent study showed that Pim-3 is involved in acquired gemcitabine resistance [18], and that it is a positive regulator of cell survival that increases the threshold for drug-induced apoptosis by upregulating the expression of survival proteins. Consistent with this, we found that miR-33a targeted Pim-3 to increase the chemosensitivity of pancreatic cancer cells and reverse the chemoresistance of pancreatic cancer cells to gemcitabine both *in vitro* and *in vivo*. High Pim-3 expression correlated with a poor prognosis, and plasma miR-33a levels were negatively correlated with the levels of Pim-3 in pancreatic tumors. Therefore, early examination of miR-33a expression in the plasma and Pim-3 expression in tissues may aid the development of new therapeutic strategies and predict the prognosis of pancreatic cancer. However, preclinical and clinical studies with large sample sizes and longer follow-up times are required to confirm the utility of these potential biomarkers.

MiR-33a inhibited the Pim-3 kinase-mediated AKT/Gsk-3β/β-catenin pathway in pancreatic cancer cells. Similarly, a previous study showed that hepatocarcinoma cancer cells overexpressing miR-33a display reduced AKT phosphorylation, which affects insulin signaling [39]. By contrast, Li's group showed that miR-33a promotes the TGF-β1-induced phosphorylation of AKT in hepatic stellate cells, which activates the cells to produce excess extracellular matrix, leading to liver fibrosis [40]. This discrepancy may be explained by the fact that a single miRNA can target multiple genes; these target genes may differ in specific cells to enable the control of different signaling pathways [37]. Because Pim-3 mRNA and protein is barely detectable in normal hepatocytes [16], miR-33a may fail to target this kinase and inhibit cell proliferation in hepatic stellate cells, which are the primary mesenchymal cells in the liver. Instead of an anti-tumor effect, the major role of miR-33a in normal cells may be to regulate lipid metabolism and/or fibrosis. By contrast, the anti-tumor effects of miR-33a might be dominant in cancer cells displaying AKT activation. MiR-33a is an essential regulator of cholesterol and lipid metabolism [41]. Hence, the findings presented here expand the biological functions of miR-33a and improve our current understanding of the relationship between metabolism and carcinogenesis.

Pim-3 can promote cell proliferation and increase gemcitabine resistance by activating AKT/β-catenin signaling. Several lines of evidence implicate Pim-3 in activating STAT3^Tyr705^ and Bad^Ser112^ phosphorylation, which leads to the expression of a number of anti-apoptotic proteins, including Bcl-X_L_ and survivin [16, 42]. Moreover, activating STAT3 increases the transcription of Hif-1α, thereby upregulating the expression of the *MDR1* (P-glycoprotein) gene, resulting in reduced chemosensitivity [43, 44]. Furthermore, other groups report that Pim-3 increases cell proliferation via p21^Cip1^, p27^Kip1^, and Cdc25C [38, 45]. Here, we found that Pim-3 stabilized and promoted the accumulation of cytoplasmic β-catenin, which upregulates cyclin D1 to promote cell-cycle progression, and upregulates the expression of survivin to inhibit apoptosis [46]. Notably, the *MDR1* gene acts downstream of β-catenin [47], suggesting that Pim-3 may contribute to MDR1 expression and gemcitabine resistance by upregulating β-catenin. Overall, we found that Pim-3 upregulated the levels of phospho-AKT, phospho-Gsk-3β, and β-catenin in gemcitabine-resistant cells; all of these molecules are associated with gemcitabine resistance [47–49]. AKT/Gsk-3β/β-catenin signaling is a component of the Wnt signaling pathway, which is involved in epithelial-mesenchymal transition [50]. Therefore, we hypothesize that Pim-3 might also induce epithelial-mesenchymal transition to mediate drug resistance.

Taken together, the results reported herein show that miR-33a is a potent tumor suppressor in the pancreas and that its growth inhibitory effects are mediated, at least in part, via downregulation of the *Pim-3* proto-oncogene. Loss of miR-33a expression, leading to the induction of Pim-3 expression, appears to be a critical event in the development of gemcitabine resistance.

## MATERIALS AND METHODS

### Human tissues and cell culture

A total of 106 human primary pancreatic adenocarcinoma tissues were collected between 2010 and 2012 at Fudan University Shanghai Cancer Center. The samples were immediately snap-frozen in liquid nitrogen and histologically examined in a timely manner. All samples were obtained with informed consent, and the project was approved by the Clinical Research Ethics Committee of Fudan University Shanghai Cancer Center.

The PCI35 and PCI55 human pancreatic carcinoma cell lines were a gift from Professor Mukaida Naofumi (Kanazawa University, Kanazawa, Japan). The SW1990, MiaPaca-2, PANC-1, BxPC-3, and Capan-1 human pancreatic carcinoma cell lines were purchased from the American Type Culture Collection (ATCC). SW1990 and Capan-1 cells were cultured in Dulbecco's Modified Eagle's Medium (Biosera), and all other cells were maintained in RPMI 1640 (Biosera). All media were supplemented with 10% FBS (Biosera), and the cells were cultured at 37°C in an incubator containing 5% CO_2_. The cell lines were authenticated by DNA profiling of short tandem repeats and amelogenin (Beijing Microread Genetics Co. Ltd.).

### Isolation of plasma miRNA

Before extracting miRNA, 5 μl of 200 pM cel-miR-39 mimics (RiboBio, Guangzhou, China) were added to 200 μl of plasma as an external standard. MiRNA was extracted from plasma using the miRcute miRNA isolation Kit (Tiangen Biotech Co. Ltd.), according to the manufacturer's instructions. Subsequently, the miRNA was reverse transcribed prior to use in quantitative reverse transcription PCR (qRT-PCR). The relative expression of target miRNA was analyzed by the ΔCt method.

### Real-time qRT-PCR

qRT-PCR was performed as described previously [38]. Detailed information is provided in the Supplementary Materials and Methods.

### Locked nucleic acid *in situ* hybridization

Locked nucleic acid *in situ* hybridization analyses of PDAC tissues were performed using a human miR-33a-specific double digoxigenin-labeled locked nucleic acid probe (Exiqon). Briefly, following deparaffinization and rehydration, tissue sections were treated with proteinase K at 37°C for 15 min and dehydrated through a graded series of ethanol solutions (70–100%). The slides were then incubated at 50°C overnight with a digoxigenin-labeled probe diluted to 250 nM in hybridization buffer. After stringent washes with 5×, 1×, and 0.2× SSC buffers at 50°C for 30 min, the slides were blocked with blocking reagent (Roche) at 37°C for 30 min and then incubated with alkaline phosphatase-conjugated anti-digoxigenin at 37°C for 30 min. Colorimetric detection was performed by incubating the slides with 4-nitro-blue tetrazolium and 5-bromo-4-chloro-30-indolylphosphate substrate (Roche) at 37°C for 120 min, followed by nuclear fast counterstain for 20 min. The tissue sections were examined in a blinded manner by a second individual. Scoring was based on the intensity of hybridization, which was designated as 0 (negative), 1 (weak), 2 (intermediate), or 3 (strong), and the percentage of positive cells, which was designated as 0 (<1%), 1 (focal, 1–30%), or 2 (diffuse, >30%). The following expression levels were based on the score obtained by multiplying the positivity and intensity scores: 0, negative (−); 1–3, weakly positive (+); 4–6, moderately positive (++); and >6, strongly positive (+++).

### Cell viability and cell cytotoxicity assays

Cell viability and cell growth inhibition were determined using Cell Counting Kit-8 (Dojindo Laboratories), according to the manufacturer's instructions. Detailed information is provided in the Supplementary Materials and Methods.

### Tumor formation assay in a nude mouse model

Eighty female BALB/c nude mice (5-weeks-old; 15–25 g) were randomly divided into eight groups. The left flank of each mouse was inoculated subcutaneously with 2 × 10^6^ MiaPaca (Mia)-2, Mia-2-miR-33a, Mia-2-res, or Mia-2-res-miR-33a cells, or with 3 × 10^6^ SW1990, SW1990-miR-33a, SW1990-res, or SW1990-res-miR-33a cells. Seven days later, each group was randomly divided into two subgroups (five mice per subgroup) and subjected to intraperitoneal injection of gemcitabine (20 mg/kg) or PBS (100 μl; negative control) every 4 days. Tumor growth was measured twice per week using calipers. Tumor volume was calculated using the following formula: (length × width^2^)/2. At Day 35 post-tumor cell injection, the tumor tissues were removed and subjected to immunohistochemical and western blot analyses. The study was performed in strict accordance with the recommendations in the Guide for the Care and Use of Laboratory Animals of Fudan University. The protocol was approved by the Committee on the Ethics of Animal Experiments of Fudan University [permit number: SYXK (Hu) 2009–0082].

### Development of gemcitabine-resistant MiaPaca-2 and SW1990 cells

MiaPaca-2 and SW1990 cells were serially sub-cultured for 9 months in medium containing incrementally increasing concentrations of gemcitabine (Sigma). The starting concentrations for the MiaPaca-2 and SW1990 cells were 0.5 μM and 0.25 μM, respectively. Both resistant cell lines (Mia-2-res and SW1990-res) retained the capacity to proliferate when cultured in medium containing gemcitabine (64 μM and 32 μM, respectively). The resistant phenotype was stable for more than 20 passages.

### Immunohistochemical analysis

Immunohistochemical staining was performed as described previously [38]. Detailed information is provided in the Supplementary Materials and Methods.

### RNA-binding protein immunoprecipitation

PCI55 and MiaPaca-2 cells were transfected with miR-33a or negative control (cel-miR-239b) mimics (RiboBio) using Lipofectamine 2000 reagent (Life Technologies), according to the manufacturer's instructions. At 48 h post-transfection, proteins were cross-linked to RNA by incubating the cells with 0.75% formaldehyde for 10 min at room temperature. The cells were then harvested in RNA-binding protein immunoprecipitation (RIP) lysis buffer [25 mM Tris-HCl, pH 7.5, 150 mM KCl, 2 mM EDTA, 0.5% NP40, 1 mM NaF, 1 mM dithiothreitol, 100 U/ml RNasin, and 1× EDTA-free protease inhibitor (Roche Diagnostics)] and centrifuged for 15 min. Half of the supernatant was collected in TRIzol reagent (Invitrogen) as an extract control, and the remainder was incubated with an anti-Argonaute1 (AGO1) antibody (1:50; Cell Signaling Technology) at 4°C overnight, followed by precipitation with 50 μl of protein G Sepharose4 Fast Flow (GE Healthcare Bio-Sciences AB) for 4 h at 4°C. After washing the beads six times with wash buffer [50 mM Tris-HCl, pH 7.5, 150 mM NaCl, 0.05% NP40, 2 mM EDTA, 1 mM dithiothreitol, and 100 U/ml RNasin (Promega)], proteinase K (Takara) was added, and the beads were incubated for 1 h at 56°C. RNA was isolated using TRIzol reagent, and qRT-PCR was performed as described above.

### Construction of Pim-3 3′UTR plasmids

The 3′UTR of *Pim-3* mRNA was amplified by PCR using human genomic DNA as a template and then subcloned into the region directly downstream of the SV40 promoter-driven *Renilla* luciferase cassette in the psiCHECK-2 vector; this vector also contains a constitutively expressed firefly luciferase gene, which was used to normalize transfection efficiency. The mutant (Δ) 3′UTR of *Pim-3*, which contained a point-mutated sequence in the seed region of miR-33a, was generated from the wild-type *Pim-3* 3′UTR plasmid by overlap-extension PCR. The structure and fidelity of the resulting constructs were confirmed by restriction mapping and sequencing. Plasmids were purified using the HighPure Maxi Plasmid Kit (Tiangen Biotech (Beijing) Co. Ltd.).

### Dual luciferase reporter assay

MiaPaca-2 and PCI55 cells were seeded into 96-well plates at a density of approximately 1.5 × 10^4^ cells/well and then co-transfected with miRNA mimics and the luciferase reporter construct using Lipofectamine 2000 reagent (Life Technologies). The cells were lysed in passive lysis buffer 48 h after transfection. The Renilla and firefly luciferase activities were quantified using the Dual Luciferase Assay System (Promega).

### Generation of Pim-3, Pim-3 short hairpin RNAs, and miR-33a stable cell lines

The procedures used to generate the stable cell lines are described in the Supplementary Materials and Methods.

### Co-immunoprecipitation and immunoblotting

Co-immunoprecipitation and immunoblotting were performed as described previously [38]. Detailed information is provided in the Supplementary Materials and Methods.

### Statistical analysis

Group comparisons of normally distributed data were performed using *t*-tests (two sample) or one-way ANOVA (multiple comparisons). The non-parametric Wilcoxon test was used to analyze continuous non-normally distributed data. For multiple comparisons, the Tukey-Kramer honestly significant difference was applied following ANOVA. Categorical variables were compared using χ^2^ analysis or Fisher's exact test. Kaplan-Meier analysis was used to analyze overall survival. *P* > 0.05 (two-tailed) was considered significant.

## SUPPLEMENTARY DATA


