# Supplementary material for: MicroRNA-33a-mediated downregulation of Pim-3 kinase expression renders human pancreatic cancer cells sensitivity to gemcitabine
Source: Oncotarget. 2015 May 8;6(16):14440–55. doi: 10.18632/oncotarget.3885 (PMC4546478; doi:10.18632/oncotarget.3885)
Supplement: Supplementary file 1 [file oncotarget-06-14440-s001.pdf]

SUPPLEMENTARY DATA

Real-time qRT-PCR

Total RNA was extracted using TRIzol reagent (Invitrogen Life Technologies) and reverse transcribed into cDNA. The qPCR analyses were performed using SYBR® Premix Ex Taq™ II (Takara Biotechnology Co. Ltd.). The relative expression levels of the target genes were determined using the ΔΔCt method. In some experiments, miRNAs were extracted from plasma samples using the miRcute miRNA Isolation Kit (Tiangen Biotech Co. Ltd.), according to the manufacturer's instructions. The miRNA was then reverse transcribed to generate the template for real-time qPCR analyses. The relative expression levels of the target miRNA were analyzed by the ΔCt method.

Generation of Pim-3 and miR-33a stable cell lines and the Pim-3 shRNA stable cell lines

The full-length wild-type human *Pim-3* cDNA was subcloned into the pMEI-5 Neo retroviral expression vector. Retrovirus production and transduction were performed as described previously [1]. The Pim-3 shRNA and non-specific control shRNA were subcloned into the lentiviral expression vector pLKO.3G. The human miR-33a sequence was subcloned into the lentiviral expression vector pCDH-CMV. Virus particles were harvested 48 h after cotransfecting HEK293T cells with the packaging plasmid ps-PAX2, the envelope plasmid pMD2.G, and the pLKO.3G-Pim-3 shRNA or pCDH-CMV-miR-33a plasmid. PCI55, SW1990, and MiaPaca-2 cells were infected with recombinant lentivirus-transducing units plus 8 µg/ml of polybrene (Sigma), and then isolated by fluorescence-activated cell sorting.

Co-immunoprecipitation and immunoblotting

Whole cell lysates were prepared in radio immunoprecipitation assay buffer containing complete protease inhibitor cocktail (Roche Diagnostics). After centrifugation for 15 min, the supernatants were incubated at 4°C overnight with 2 µg of a rabbit anti-Pim-3 antibody or 2 µg of a rabbit anti-Gsk-3β antibody, and then precipitated with 20 µl of protein GSepharose 4 Fast Flow (GE Healthcare) for 2 h at 4°C. After washing with cell lysis buffer, the materials bound to the beads were eluted with SDS-PAGE loading buffer, separated on 10% SDS-PAGE gels, and transferred onto a polyvinylidene difluoride membrane (Millipore). After soaking with 3% bovine serum albumin, the membrane was incubated with a rabbit monoclonal anti-Pim-3 or rabbit anti-Gsk-3β antibody (1:1000; Cell Signaling Technology). In some experiments, the membrane was incubated with the following antibodies: rabbit monoclonal anti-phospho-Akt^{Ser473}, mouse monoclonal anti-AKT, rabbit monoclonal anti-non-phospho-β-catenin, rabbit monoclonal anti-phospho-Gsk-3β (all 1:1000;Cell Signaling Technology), or rabbit monoclonal anti-actin (1:5000; Sigma). The membrane was then incubated with ImmunoPure peroxidase-conjugated anti-rabbit IgG or anti-mouse IgG. The blotted membrane was treated with SuperSignal West Dura Extended Duration Substrate, and the signals were detected using a LAS-4000 mini CCD camera. The expression levels of Pim-3 and actin were quantified using the NIH ImageJ software. The Pim-3/actin ratios were calculated for each cell line.

Cell viability and cytotoxicity assays

Cells were seeded into a 96-well plate at a density of 2×10^3 cells/well; After 12 h, when the cells were adhered to the microplate, was designated as time 0. Cell viability was determined every day using Cell Counting Kit-8 (Dojindo Laboratories), according to the manufacturer's instructions, and the viability ratios were calculated with reference to day 0. For cell cytotoxicity assays, cells were seeded into 96-well plates at a density of 1×10^4 cells/well, and then exposed to different concentrations of gemcitabine for 48 h, or the same dose (200 nM for SW1990, 350 nM for MiaPaca-2, 30 µM for SW1990-res, 60 µM for MiaPaca-2-res cells) for different times. Inhibition of cell growth was determined from three separate experiments using Cell Counting Kit-8. The concentration of gemcitabine that inhibited 50% of the cell viability was extrapolated from non-linear least squares curve fitting of the dose-response curves (GraphPad PRISM, Intuitive Software for Science), and then used to obtain IC_{50} values.

Immunohistochemical analyses of human pancreatic cancer tissues

Immunohistochemical staining of 4 µm sections of paraffin-embeddedtissues was performed to determine the expression level of Pim-3 protein. Briefly, the slides were incubated with a rabbit anti-Pim-3 antibody (1:100; Cell Signaling Technology), followed by goat anti-rabbit IgG. The Pim-3 immunoreactivity was visualized using the GT vision DAB kit (GeneTech Co. Ltd.). The slides were counterstained with ChemMateHematoxylin (Dako Cytomation) and observed under a microscope (Olympus). The proportions of Pim-3-positive cells in human pancreatic carcinoma tissues were evaluated by

a pathologist who did not have prior knowledge of the clinical information. Scoring was based on the percentage of positively-stained cells, which was designated as 0 (<5% of total cells), 1 (5–25%), 2 (25–50%), or 3 (>50%), and the intensity of the staining, which was designated as 0 (no coloration), 1 (pale yellow), 2 (yellow), or 3 (claybank). The following Pim-3 expression levels were classified based on the score obtained by multiplying the positivity and intensity scores: 0, negative (−); 1–3, weakly positive (+); 4–6, moderately positive (++); and >6, strongly positive (+++).

REFERENCES

1. Liu B, Wang Z, Li HY, Zhang B, Ping B, Li YY. Pim-3 promotes human pancreatic cancer growth by regulating tumor vasculogenesis. Oncol Rep. 2014; 31:2625–2634.

A

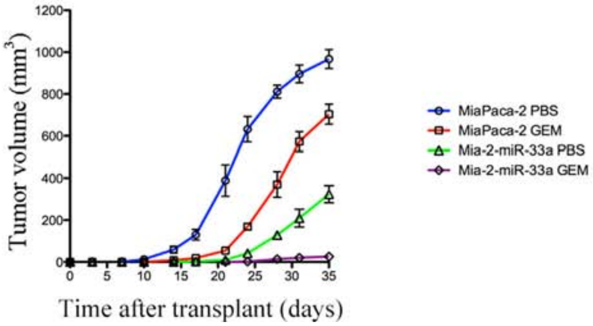

B

	Mia-2		Mia-2-miR-33a	
	PBS	GEM	PBS	GEM
**# Mice**	5	5	5	5
**Tumorigenic Mice**	5	5	3	2
**Tumorigenic ability**	100%	100%	60%	40%

Supplementary Figure S1: Overexpression of miR-33a could increase MiaPaca-2 pancreatic cancer cells chemosensitivity to gemcitabine *in vivo*. A. Stable expression of miR-33a in MiaPaca-2 cells synergistically enhanced gemcitabine to inhibit tumor growth in the xenograft nude mouse model. Tumor sizes were measured twice a week. The mean and SEM were calculated and are shown here. **B.** Incidence of tumor formation from MiaPaca-2 or MiaPaca-2-miR-33a cells with PBS or gemcitabine treat on day 35 was determined.

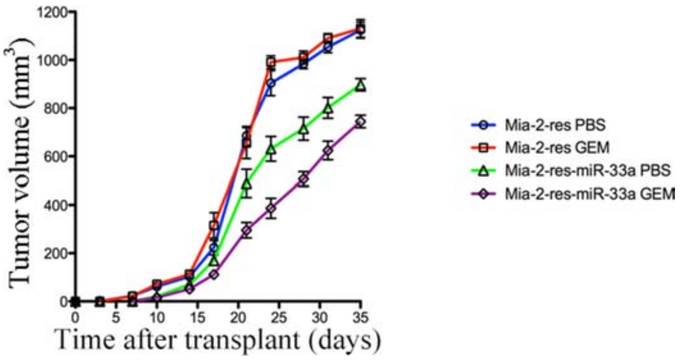

Supplementary Figure S2: Overexpression of miR-33a could reverse pancreatic cancer gemcitabine chemoresistance *in vivo*. Stable expression of miR-33a in MiaPaca-2 resistance cells significantly reversed gemcitabine resistance to inhibit tumor growth in the xenograft nude mouse model. Tumor sizes were measured twice a week. The mean and SEM were calculated and are shown here.

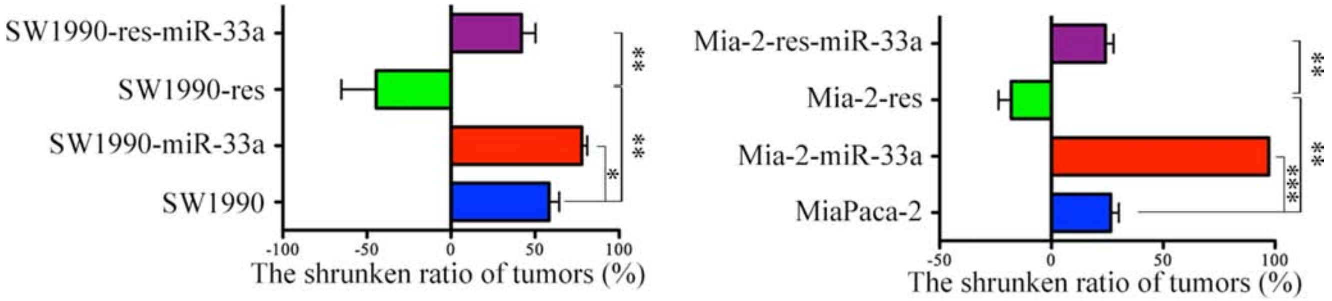

Supplementary Figure S3: The shrunken ratio of tumors in SW1990 (left) and MiaPaca-2 (right) parental cells, resistance cells, and miR-33a stable expression cells treatment with gemcitabine were calculated. The decreased tumor volumes were relative to the parental cell group treated with PBS. The results are presented as the mean +/− SEM. Two-tailed Student's *t* test was performed for statistical analysis, $*P < 0.05$, $**P < 0.01$ and $***P < 0.001$.
